# Structural polymorphism of the antigenic loop in HBV surface antigen dictates binding of diverse neutralizing antibodies

**DOI:** 10.1038/s41421-025-00803-2

**Published:** 2025-06-17

**Authors:** Xiao He, Weiyu Tao, Yunlu Kang, Jiaxuan Xu, Xiaoyu Liu, Lei Chen

**Affiliations:** 1 State Key Laboratory of Membrane Biology, College of Future Technology, Institute of Molecular Medicine, Beijing Key Laboratory of Cardiometabolic Molecular Medicine, Peking University, Beijing, China; 2 Academy for Advanced Interdisciplinary Studies, Peking University, Beijing, China; 3 Peking-Tsinghua Center for Life Sciences, Peking University, Beijing, China; 4 National Biomedical Imaging Center, Peking University, Beijing, China

**Keywords:** Electron microscopy, Immunology

## Abstract

The Hepatitis B Virus (HBV) poses a significant health threat, causing millions of deaths each year. Hepatitis B surface antigen (HBsAg), the sole membrane protein on the HBV viral envelope, plays crucial roles in viral attachment to host cells and serves as the target for neutralizing antibodies (NAbs). Despite its functional and therapeutic significance, the mechanisms by which NAbs recognize HBsAg remain elusive. Here, we found that HBsAg proteins exist in distinct subtypes and are recognized by different groups of antibodies. Cryo-electron microscopy (Cryo-EM) structures of HBsAg dimers in complex with NAb Fab fragments reveal that the antigenic loop (AGL) of these distinct HBsAg types share a common structural core comprised of four β-strands. However, their surface structures exhibit unexpected polymorphism due to distinct disulfide bond linkages within the AGL region. This structural polymorphism determines the recognition of HBsAg by different groups of NAbs.

## Introduction

Hepatitis B is a viral infection that affects the liver and can lead to both acute and chronic diseases^1^. The World Health Organization (WHO) estimates that in 2022, ~254 million people were living with chronic hepatitis B infection, with 1.2 million new cases occurring each year. In the same year, hepatitis B was responsible for an estimated 1.1 million deaths, primarily due to cirrhosis and hepatocellular carcinoma (primary liver cancer).

HBV is encased in a viral envelope that contains HBsAg as its only protein component^2^. There are three isoforms of HBsAg — large (L-HBsAg), medium (M-HBsAg), and small (S-HBsAg) surface antigens — resulting from alternative translational starting sites^3^. L-HBsAg is the longest ORF of HBsAg and contains PreS1, PreS2, and S-HBsAg, while the M-HBsAg contains PreS2 and S-HBsAg^3^. S-HBsAg is a transmembrane protein with a cytosolic loop (CYL) and an extracellular hydrophilic antigenic loop (AGL) or the “a”-determinant^4^, which contains the B cell epitope recognized by the host adaptive humoral immunity.

The process of HBV attachment begins with low-affinity interactions between the AGL of HBsAg on the virus envelope and heparan sulfate proteoglycans on the surface of hepatocytes^5^. Following this initial attachment, high-affinity interactions occur between the PreS1 region of L-HBsAg and its specific receptor, sodium taurocholate cotransporting polypeptide (NTCP), located on the plasma membrane of hepatocytes^6^. Notably, the Hepatitis D Virus also uses HBsAg as its envelope protein and shares the same entry pathway as HBV^7^. Studies have demonstrated that antibodies targeting either the AGL or the PreS1 region exhibit neutralizing activity^8^. For several decades, polyclonal hepatitis B immunoglobulins (HBIG) have been effective in post-exposure prophylaxis (PEP) against HBV infection, particularly in neonates born to HBsAg carrier mothers and in liver transplant patients with HBV infection^9,10^. Various neutralizing antibodies (NAbs) targeting AGL are currently being evaluated in clinical trials for their potential use in PEP or the treatment of chronic Hepatitis B^8^. The crucial role of AGL in HBV infection is evident from the fact that hepatitis B can be effectively prevented through vaccines containing the S-HBsAg protein, which exposes AGL as the major epitope^8^. Therefore, the AGL on the S-HBsAg is the primary target of broad NAbs^11^ and is also the site where escape mutations often occur^11,12^, such as the well-known G145R mutation^13^.

HBsAg dimer is the basic building block of the viral envelope. The core structure of HBsAg dimer only recently emerged from structures determined from the spherical subviral particles (SVP)^14–16^. Particularly, our studies showed that the CYL of each HBsAg has a zinc finger motif, and the HBsAg dimer interface involves interactions between H1, CYL, and H2 of two protomers^16^. However, the structure of AGL is missing in these studies due to its poor local resolution. Up to today, there is only one structure of a short peptide of AGL in complex with an NAb (H015) available^11^. Therefore, the structure of the essential AGL region and how it is recognized by NAbs remain largely enigmatic. Here, we combine biochemical assays and structural determination, uncovering the structural polymorphism of the AGL and the binding mechanism of HBV NAbs.

## Results

### Diverse NAbs recognize distinct forms of HBsAg

To study the interactions between HBsAg and NAbs, we gathered sequences of high-affinity HBV NAbs that bind to the AGL from the literature and identified NAb_H006_^11^, NAb_H015_^11^, NAb_HBC_ (the parental antibody of VIR-3434)^17^, and NAb_GC1102_^18^ for our investigations. To obtain the HBsAg protein for biochemical characterization, we appended a signal peptide to the N-terminus of a GFP-tagged M-HBsAg construct. This modified protein demonstrated a strong interaction with Fab_HBC_, indicating that its AGL was correctly folded (Supplementary Fig. S1a). To reduce the aggregation of HBsAg, we introduced alanine mutations at three cysteines (C76A, C90A, C221A) in M-HBsAg. These cysteines are not conserved in other HBV-related viruses and do not affect the surface expression of HBsAg^16^. The resulting M-HBsAg-3CA construct also binds strongly to Fab_HBC_ (Supplementary Fig. S1a), confirming that HBsAg-3CA maintains a correctly folded AGL and is suitable for further biochemical and structural characterization.

**Fig. 1:**
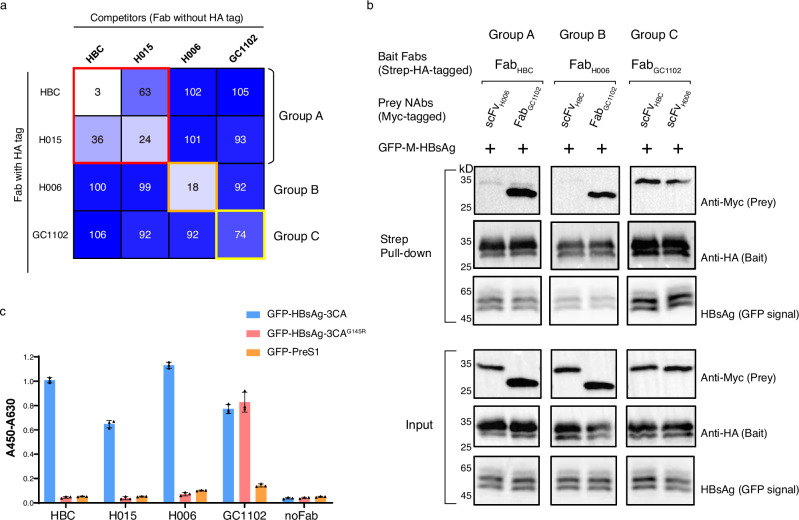
Classification of NAbs targeting AGL of HBsAg. **a** Heat map illustrating the competitive binding behaviors of four neutralizing antibodies (NAbs) for HBsAg: NAb_HBC_, NAb_H006_, NAb_H015_, and NAb_GC1102_. The binding of HA-tagged antibodies in the presence of non-tagged competitors, as determined by ELISA, is normalized to their binding without competitors. Numbers are represented with background colors, where darker shades indicate higher values and less competition. Data are presented as mean, with *n* = 3 technical replicates. The experiment was conducted twice with consistent results. **b** Strep-tag pull-down experiments were conducted to assess the binding compatibilities of NAbs across different groups. HA-tagged Fabs containing Strep tags were bound to Streptactin resin as bait. GFP-tagged M-HBsAg was added to mediate the binding between compatible NAbs. Myc-tagged Fabs or scFv served as prey. Proteins were detected using western blotting (for HA or Myc tags) or in-gel fluorescence imaging for GFP (M-HBsAg). **c** Binding of NAbs to HBsAg and its G145R mutant was assessed using ELISA. GFP-PreS1 was used as the negative control to assess the non-specific binding. Data are shown as mean ± standard deviations, *n* = 3 technical replicates. The experiment was performed twice with consistent results.

To investigate whether these NAbs bind to the same epitope on HBsAg, we performed a competitive ELISA (Fig. 1a) and found that NAb_HBC_ and NAb_H015_ belong to one group (Group A), while NAb_H006_ and NAb_GC1102_ are categorized into two other groups (Group B and Group C, respectively). NAbs within each group exhibit competitive binding behavior, whereas NAbs from different groups are non-competitive. This suggests that NAbs in different groups may recognize distinct epitopes on the same type of HBsAg or markedly different types of HBsAg.

To further distinguish these possibilities, we conducted ternary pull-down experiments using Fab_HBC_, Fab_H006_, and Fab_GC1102_ as representatives for Groups A, B, and C, respectively. We employed Fab_HBC_ with strep and HA tag to pull down Myc-tagged scFv_H006_ or Fab_GC1102_ in the presence of GFP-M-HBsAg. The results demonstrated that NAb_HBC_ and NAb_GC1102_ can bind to HBsAg simultaneously, while NAb_HBC_ and NAb_H006_ cannot. Additional pull-down experiments with either strep-tagged Fab_H006_ or Fab_GC1102_ confirmed that NAb_GC1102_ and NAb_H006_ can also bind to HBsAg simultaneously, while NAb_HBC_ and NAb_H006_ cannot bind to HBsAg at the same time (Fig. 1b). These compelling biochemical results support a model in which Fab_HBC_ and Fab_H006_ bind to distinct types of HBsAg (Type A and Type B, respectively), while Fab_GC1102_ binds to both Type A and Type B. These unexpected findings suggest that HBsAg of Types A and B may exhibit different structures.

Additionally, we found that NAbs in Group A (Fab_HBC_ and Fab_H015_) and Group B (Fab_H006_) almost lost their binding to G145R escape mutant (Fig. 1c). In contrast, the NAb in Group C (Fab_GC1102_) retains robust binding to HBsAg despite the G145R mutation (Fig. 1c). This agrees with previous results on the neutralizing effects of these NAbs against escape mutations^11,17,18^ and suggests that NAbs in Group C bind to a unique epitope on HBsAg.

### AGL_Type A_ and AGL_Type B_ show distinct structures with different disulfide linkages

**Fig. 2:**
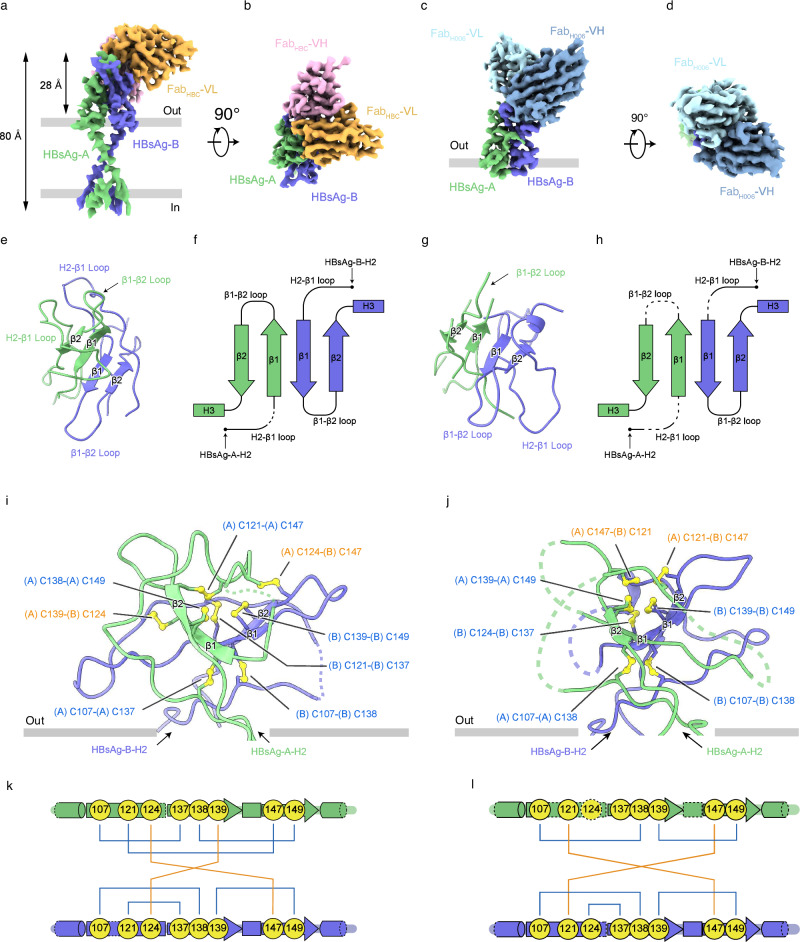
The structures of HBsAg_Type A_ and HBsAg_Type B_. **a** Side view of the cryo-EM map of the HBsAg–Fab_HBC_ complex. HBsAg-A, HBsAg-B, Fab_HBC_-heavy chain variable region (VH), and Fab_HBC_-light chain variable region (VL) are colored in green, purpleblue, pink, and orange, respectively. The sizes of the HBsAg and AGL are labeled. The detergent micelle and unresolved flexible region are shown in semi-transparent gray. The approximate boundaries of the phospholipid bilayer are indicated as thick gray lines. **b** A 90° rotated top view of **a**. **c** Side view of the cryo-EM map of the HBsAg–Fab_H006_ complex. HBsAg-A subunit and HBsAg-B subunits are colored the same as in (**a**), while Fab_H006_-VH and Fab_H006_-VL are colored in steel blue and light blue, respectively. The approximate boundaries of the outer leaflet of the viral envelope are indicated as thick gray lines. **d** A 90°-rotated top view of **c**. **e** Top view of the AGL_Type A_ in cartoon representation. AGL-A and AGL-B are in the same colors as in (**a**). β-strands and loops are indicated. **f** 2D topology diagram of AGL_Type A_. β-strands and loops are indicated. Flexible regions that are not resolved are denoted with dashed lines. **g** Top view of the AGL_Type B_ in cartoon representation. AGL-A and AGL-B are in the same colors as in (**c**). **h** 2D topology diagram of AGL_Type B_. **i** Side view of the AGL_Type A_ in cartoon representation. Cysteine pairs forming disulfide bonds are indicated, and the disulfide bonds are highlighted in yellow. **j** Side view of the AGL_Type B_ in cartoon representation. **k** Schematic diagram of AGL_Type A_. Cysteines are indicated by yellow circles with their residue numbers. The intramolecular disulfide bonds are labeled in blue, and the intermolecular disulfide bonds in orange. Flexible regions that are not resolved are denoted with dashed lines. **l** Schematic diagram of AGL_Type B_.

To understand the structures of two types of HBsAg and how they are recognized by different groups of antibodies, we determined the structure of HBsAg_Type A_ in complex with Fab_HBC_ and HBsAg_Type B_ in complex with Fab _H006_ at resolutions of 3.25 Å and 3.13 Å respectively (Fig. 2; Supplementary Figs. S1b–f, S2–S5, and Table S1). In both cases, we found that the Fabs bind to HBsAg dimer in 1:1 stoichiometry (Fig. 2a–d).

In the map of HBsAg_Type A_ in complex with Fab_HBC_, we could resolve the density of a portion of the H1, H2, AGL, and H3 regions of HBsAg (Fig. 2a, b; Supplementary Fig. S2), but not other regions, suggesting that they have considerable flexibility in detergent micelles. The partially disordered transmembrane region is likely due to the loss of contacts between HBsAg dimers during detergent solubilization, as observed in previous studies of the detergent-solubilized spherical SVP^14^. Further masking out the transmembrane helices and detergent micelle improved the resolution of the AGL-Fab region to 3.20 Å (Supplementary Fig. S2 and Table S1). The structure of HBsAg_Type A_ shows that the anti-parallel β1 and β2 strands of two HBsAg subunits pack together to form a central four-strand β-sheet core of AGL (Fig. 2e, f). The long H2-β1 loop and β1-β2 loop are exposed to the solvent and contribute to the majority of the surface properties of the AGL region (Fig. 2e). The AGL region of each HBsAg subunit contains eight cysteines, and thus the AGL of HBsAg dimer contains 16 cysteines in total (Fig. 2i, k). To our surprise, although the 16 cysteines in the AGL_Type A_ form six intramolecular disulfide bonds and two intermolecular disulfide bonds (Fig. 2i, k), the cysteine pairs that form these disulfide bonds are completely different between the two HBsAg subunits in HBsAg_Type A_ (Fig. 2i, k). For example, C107 pairs with C137 in subunit A, while C107 pairs with C138 in subunit B (Fig. 2i, k; Supplementary Fig. S3a). To our knowledge, such a drastically asymmetric disulfide linkage has not been reported in any homodimeric protein previously. The asymmetric disulfide bonds confer structural asymmetry to the AGL_Type A_. Close inspection reveals that the symmetry breakage occurs in the AGL, starting around C107 where the first disulfide bond is formed, and ending around C149 where the last disulfide bond is formed (Supplementary Fig. S3d, e).

In the map of HBsAg_Type B_ in complex with Fab_H006_ (Fig. 2c, d; Supplementary Figs. S4, S5 and Table S1), one AGL protomer bound to Fab_H006_ showed better density than the other, although some residues on AGL remained unresolved due to their flexibility (Fig. 2c, d, g, h). Compared to AGL_Type A_, AGL_Type B_ adopts a distinct conformation, which also features a structural core formed by four anti-parallel β-strands at its center, surrounded by loop-like structures composed of connecting residues (Fig. 2g, h). We found that the 16 cysteines in AGL form five intramolecular disulfide bonds and two intermolecular disulfide bonds, with the side chains of C124 and C137 remaining flexible (Fig. 2j, l). Notably, during model building, we observed connecting densities between two adjacent disulfide bonds (between the C139–C149 bonds of each protomer), suggesting a degree of heterogeneity in disulfide linkages (Supplementary Fig. S5). The disulfide linkages in AGL_Type B_ are dramatically different from those in AGL_Type A_ (Fig. 2k, l).

### Mechanism of AGL_Type A_ and AGL_Type B_ recognition by NAbs

**Fig. 3:**
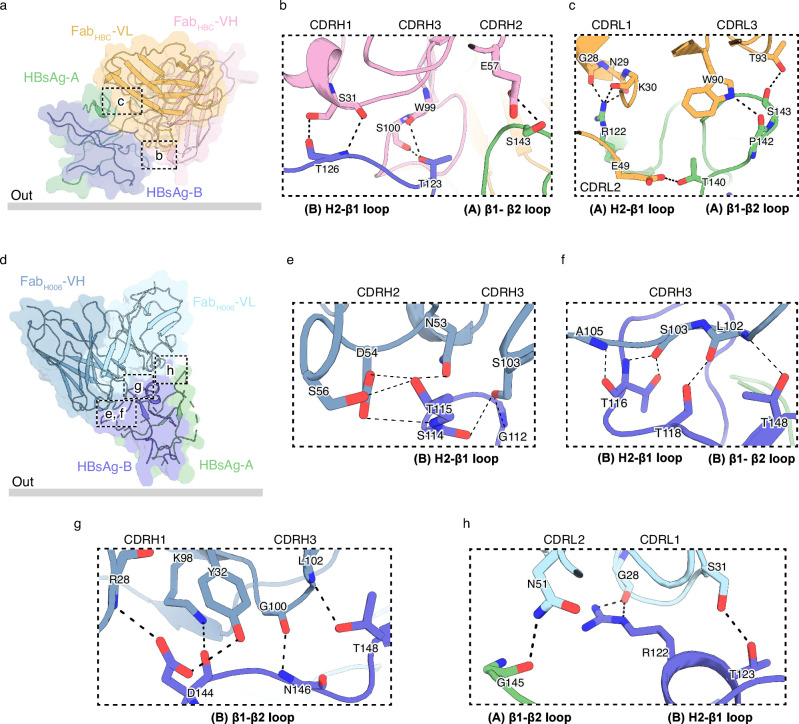
Interactions between AGL and different Fabs. **a** Side view of AGL_Type A_ and Fab_HBC_-Fv domains in a semi-transparent surface representation, along with the atomic model in the same color as in Fig. 2a. The viral envelope membrane is indicated with a thick gray line. Interfaces are highlighted with dashed boxes. **b** Close-up view of the interface between Fab_HBC_-VH and HBsAg boxed in (**a**). Key residues for the binding between Fab_HBC_-VH and HBsAg are shown as sticks. **c** Close-up view of the interface between Fab_HBC_-VL and HBsAg boxed in (**a**). Key residues for the binding between Fab_HBC_-VL and HBsAg are shown as sticks. **d** Side view of AGL_Type B_ and Fab_H006_-Fv domains in a semi-transparent surface representation, along with the atomic model in the same color as in Fig. 2c. The viral envelope membrane is indicated with a thick gray line. Interfaces are highlighted with dashed boxes. **e**–**g** Close-up views of the interfaces between Fab_H006_-VH and HBsAg boxed in (**d**). Key residues for the binding between Fab_H006_-VH and HBsAg are shown as sticks. **h** Close-up views of the interfaces between Fab_H006_-VL and HBsAg boxed in (**d**). Key residues for the binding between Fab_H006_-VL and HBsAg are shown as sticks.

The structure of HBsAg_Type A_ in complex with Fab_HBC_ shows that Fab_HBC_ binds to one side of AGL through an extended interface (Fig. 3a). Both the heavy chain and the light chain of Fab_HBC_ contribute to the binding to a structural epitope on the surface of AGL, which is shaped by the H2-β1 loop and the β1-β2 loop on subunit A and the H2-β1 loop on subunit B (Fig. 3a). Particularly, the H2-β1 loop of subunit B is recognized by CDRH1 and CDRH3 of Fab_HBC_ and the H2-β1 loop of subunit A is bound by CDRL1 of Fab_HBC_ (Fig. 3b, c). The β1-β2 loop on subunit A binds to CDRH2 and CDRL3 of Fab_HBC_ (Fig. 3b, c). Detailed polar interactions between HBsAg and Fab_HBC_ are listed in Supplementary Table S2.

The structure of HBsAg_Type B_ in complex with Fab_H006_ shows that Fab_H006_ binds to the top of AGL_Type B_ on one side (Fig. 3d), primarily interacting with one subunit of HBsAg. Both the heavy and light chains of Fab_H006_ contribute to AGL recognition (Fig. 3e, h). The H2-β1 loop on AGL is bound by CDRL1, CDRH2, and CDRH3 of Fab_H006_ (Fig. 3e, h). The β1-β2 loop binds to CDRH1 and CDRH3 of Fab_H006_ (Fig. 3e, h). Detailed polar interactions between HBsAg and Fab_H006_ are listed in Supplementary Table S3.

Structural alignment of AGL_Type B_ with AGL_Type A_ using the central four β-strands revealed that the structural epitopes recognized by NAb_H006_ and NAb_HBC_ are strikingly distinct (Supplementary Fig. S6). Notably, due to the different disulfide bond linkages, the structural epitope of NAb_HBC_ on AGL_Type A_ does not exist in AGL_Type B_, and vice versa (Supplementary Fig. S6), which agrees with the high cross-group specificity of NAbs in Groups A and B (Fig. 1).

### NAb_GC1102_ in Group C binds to a unique epitope shared by HBsAg_Type A_ and HBsAg_Type B_

**Fig. 4:**
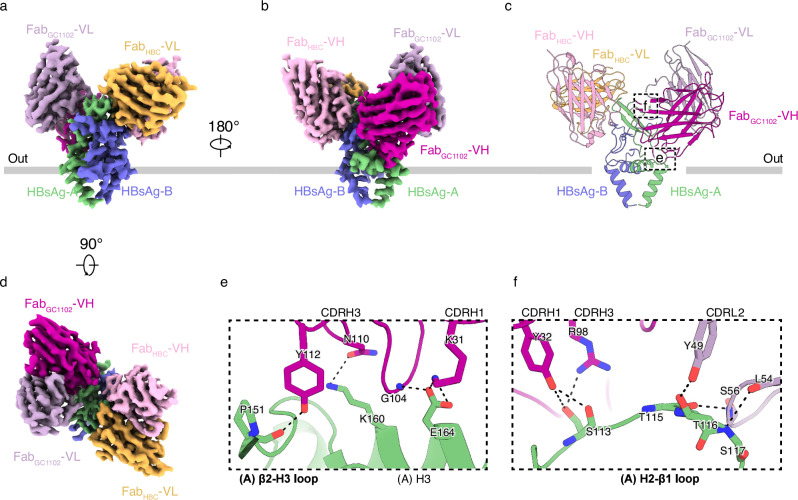
The structure of AGL_Type A_ in complex with Fab_GC1102_ and Fab_HBC_. **a** Side view of the cryo-EM map of the HBsAg–Fab_GC1102_–Fab_HBC_ complex. Fab_GC1102_-VH and Fab_GC1102_-VL are colored in purple and light purple, respectively, and others are colored the same in Fig. 2a. The approximate boundaries of the outer leaflet of the viral envelope are indicated as dashed lines. **b** A 180°-rotated side view of **a**. **c** Atomic model of the HBsAg-Fab_GC1102_-Fab_HBC_ complex is shown as cartoons in the same colors as in (**b**). Locations of interactions between Fab_GC1102_ and HBsAg are indicated with dashed boxes. **d** A 90°-rotated top view of **a**. **e** Close-up view of the interface between Fab_GC1102_-VH and HBsAg boxed in (**c**). Key residues for the binding between Fab_GC1102_-VH and HBsAg are shown as sticks. **f** Close-up view of the interface between Fab_GC1102_ and HBsAg H2-β1 loop boxed in (**c**). Key residues for the binding between Fab_GC1102_ and HBsAg H2-β1 loop are shown as sticks.

NAb_GC1102_, a Group C NAb, can bind to both HBsAg_Type A_ and HBsAg_Type B_, indicating that it recognizes a common epitope shared by both types of HBsAg proteins. To elucidate the underlying mechanism, we determined the structure of HBsAg in complex with both Fab_HBC_ and Fab_GC1102_ to the resolution of 3.09 Å (Fig. 4; Supplementary Figs. S1g–h, S7, and Table S1). The structure reveals that one Fab_GC1102_ binds to the opposite side of Fab_HBC_, engaging the lower region of one HBsAg _Type A_ subunit (Fig. 4a–d). The epitope recognized by Fab_GC1102_ includes the H2-β1 loop, β2-H3 loop, and H3 of HBsAg subunit A (Fig. 4e, f). β2-H3 loop and H3 are bound by CDRH3 and CDRH1, while H2-β1 loop interacts with CDRH1, CDRH3, and CDRL2 (Fig. 4e, f). Detailed polar interactions between HBsAg and Fab_GC1102_ are listed in Supplementary Table S4.

Further structural modeling suggests that the epitope recognized by Fab_GC1102_ is also present in HBsAg_Type B_ (Supplementary Fig. S7h, i). Additionally, both Fab_GC1102_ and Fab_H006_ can bind to HBsAg_Type B_ simultaneously without sterical clashes, consistent with our biochemical results (Supplementary Fig. S7h, i).

## Discussion

The structures of the AGL region of HBsAg reveal a remarkable and unexpected structural polymorphism. Our studies identified three distinct groups of NAbs, closely aligning with findings from a comprehensive study involving 144 vaccinated or HBV-infected individuals^11^. By mapping our results to theirs using the common NAbs H015 and H006, we found that Groups A and B are the largest NAb groups identified in humans^11^. This analysis suggests that Type A and B HBsAg may represent the predominant forms of HBsAg on the viral surface, while the existence of other forms remains elusive. Additionally, the cysteines forming the disulfide bonds are highly conserved in the surface proteins of HBV-related viruses (Supplementary Fig. S8). This conservation leads us to speculate that these viral surface proteins may exhibit similar structural polymorphisms to those observed in HBsAg. Our structures also elucidate how Types A and B HBsAg are recognized by their respective NAbs. Notably, numerous naturally occurring HBV escape mutations have been documented, including the well-known G145R mutation^13^. Many of these mutations occur within the epitopes of NAb_HBC_ and NAb_H006_^13^ (Supplementary Figs. S9, S10). However, only one mild mutation, T126S, is found within the epitope of NAb_GC1102_ (Supplementary Figs. S9, S10), likely because that NAb_GC1102_ binds to the extracellular lower region of HBsAg. This unique binding mode correlates with its broad neutralizing activity against several common escape mutations^18^, whereas NAb_HBC_ and NAb_H006_ are more sensitive to specific escape mutations like G145R^11,17^. Furthermore, these structures provide insights into how the human humoral response generates diverse antibodies that recognize distinct forms of HBsAg, as well as different epitopes on the same form (Supplementary Fig. S11). These mechanistic insights lay the foundation for developing combination therapies that utilize multiple NAbs targeting different epitopes on distinct types of HBsAg for the treatment of HBV infection.

## Materials and methods

### Cell culture

Sf9 insect cells (Thermo Fisher Scientific) were cultured in SIM SF (Sino Biological) at 27 °C. FreeStyle 293 F suspension cells (Thermo Fisher Scientific) were cultured in FreeStyle 293 medium (Thermo Fisher Scientific) supplemented with 1% fetal bovine serum (FBS, VisTech), 67 μg mL^–1^ penicillin (Macklin), and 139 μg mL^–1^ streptomycin (Macklin) at 37 °C with 6% CO_2_ and 70% humidity. The cell lines were routinely checked to be negative for mycoplasma contamination but have not been authenticated.

### Constructs

Superfolder-GFP-tagged M-HBsAg was generated by fusing the coding sequence of the GFP superfolder before the preS2 region of M-HBsAg (serotype ayw, genotype D3), with a PreScission Protease cleavage sequence between them. A rat FSHβ signal peptide sequence was inserted at the N-terminus of the GFP. The ORF was cloned into a modified BacMam vector^19^. Point mutations on M-HBsAg were introduced using Quikchange PCR.

The coding sequences of the variable heavy (V_H_) and light chains (V_L_) of Fabs obtained from literature and patents were synthesized and cloned into a pBMCL1-based plasmid^20^ and fused with the human CH1 region and human kappa CL region, respectively. The 3× HA-tagged Fab was generated by introducing a 3× HA tag at the C-terminus of the light chain. The coding sequence of the anti-human kappa nanobody^21^ was synthesized and cloned into pET26b, with a tandem 6× His-twin Strep-FLAG tag at the C-terminus.

For Fab_H006_, V11 and P118 on the VH, P158 on CH1, P39 and A79 on VL, and E166 and S172 on the kappa CL region were mutated to cysteine. In addition, a cysteine was inserted between E159 and P160 on the CH1 region. These eight cysteines were expected to generate four disulfide bonds (4DS) between the variable and constant regions of the Fab fragment to stabilize its conformation^22^.

For Fab_GC1102_, L11 and T124 on the VH, P163 on CH1, P40 and P80 on VL, and E165 and S171 on the kappa CL region were mutated to cysteine. In addition, a cysteine was inserted between E164 and P165 on the kappa CL region. These eight cysteines were expected to generate four disulfide bonds between the variable and constant regions of the Fab fragment to stabilize its conformation.

### Expression and purification of Fab_HBC,_ Fab_GC1102_, and Fab_H006_

Fab_HBC_, Fab_GC1102_, and Fab_H006_ were purified similarly. Briefly, FreeStyle 293F cells at a density of around 2.5 × 10^6^ cells mL^–1^ were co-transfected with constructs containing the heavy chain and the light chain of Fab at a ratio of 1:1 using polyethyleneimine (PEI-MAX, Polysciences). 10 mM sodium butyrate was added 12 h after transfection and the cells were incubated at 37 °C for 108 h. Cell pellets were removed by centrifugation at 3993× *g* for 20 min at 4 °C (Beckman JA8.1), and the supernatant was subjected to Ni-NTA affinity chromatography. The column was washed tandemly with W buffer 1 (50 mM Tris (pH 7.5), 500 mM NaCl, and 10 mM imidazole), W buffer 2 (20 mM Tris (pH 7.5), 150 mM NaCl, and 20 mM imidazole), and W buffer 3 (20 mM Tris (pH 7.5), 50 mM NaCl and 30 mM imidazole). Fab was eluted with elution buffer containing 300 mM imidazole (pH 8.0) and 25 mM NaCl. The pH of the eluate was adjusted to 6.0 with 10% glacial acetic acid and SP-A buffer (20 mM MES, pH 6.0) was added into the eluate until the conductance was under 5000 μS cm^–1^. The Fab was loaded onto an SP HP 5 mL column (Cytiva) and eluted with SP-B (20 mM MES, pH 6.0, 1 M NaCl) in a linear gradient using the AKTA pure system (GE Healthcare). Fractions containing Fabs were collected and supplemented with 10% glycerol, flash frozen, and stored at –80 °C.

### ELISA

To estimate the binding affinity between HBsAg and Fabs, ELISA was performed using purified M-HBsAg and Fabs. Briefly, 12-well strips (Yunpeng) were coated with anti-GFP nanobody^23^, then blocked by TBSG-FBS (20 mM Tris (pH 7.5), 150 mM NaCl, 0.0058% GDN, and 2% FBS) for 30 min. 50 nM GFP-tagged M-HBsAg (WT or 3CA) in TBSG (20 mM Tris (pH 7.5), 150 mM NaCl, 0.0058% GDN) was added (100 μL per well) and incubated on ice for 1 h to bind anti-GFP nanobody. An unrelated protein, GFP-tagged anti-ALFA nanobody^24^ was used as the negative control. Unbounded antigens were washed away by TBSG before the addition of different concentrations of 3× HA-tagged Fab_HBC_ (50 μL per well in TBSG-FBS) for 1 h on ice. After washing with TBSG, rabbit anti-HA (3724; CST, diluted 4000 times in TBSG-FBS, 50 μL per well) was added and incubated on ice for 1 h to bind 3× HA-tagged Fab. After washing with TBSG, goat anti-rabbit antibody conjugated with horseradish-peroxidase (HRP) (31460; Thermo Fisher Scientific, diluted 4000 times in TBSG-FBS, 50 μL per well) was added and incubated on ice for 1 h. After extensive washing with TBSG, 100 μL of ELISA developing buffer (51.5 mM Na_2_HPO_4_, 24.3 mM citric acid, 0.006% H_2_O_2_, and 0.1 mg mL^−1^ 3,3′,5,5′-tetramethylbenzidine (TMB)) was added and incubated at room temperature for 10 min before stopping with 100 μL 2 M H_2_SO_4_. ELISA signals were measured by absorbance at 450 nm with an Infinite M Plex plate reader (Tecan).

To assess the binding capability of Fabs to HBsAg G145R mutant, 50 nM GFP-tagged M-HBsAg (3CA or 3CA^G145R^) and GFP-PreS1 proteins were added to anti-GFP nanobody-coated strips. 3× HA-tagged Fabs (50 μL per well in TBSG-FBS) were added and incubated on ice for 1 h. After washing with TBSG, rabbit anti-HA (3724; CST, diluted 4000 times in TBSG-FBS, 50 μL per well) was added and incubated on ice for 1 h to bind 3× HA-tagged Fab. The following steps were the same as described above.

For competitive ELISA, GFP-M-HBsAg-3CA was immobilized on anti-GFP nanobody-coated strips as described above. The antigen was incubated with 200 nM of different Fabs (50 μL per well, for GC1102, the concentration was 400 nM) for 2 h at 4 °C to block their structural epitope on HBsAg. After washing with TBSG, HA-tagged Fabs (50 μL per well) were added and incubated on ice for 30 min. Empty buffer was used as the negative control. After washing with TBSG, rabbit anti-HA (3724; CST, diluted 4000 times in TBSG-FBS, 50 μL per well) was added and incubated on ice for 1 h to bind HA-tagged Fabs. The following steps were the same as described above.

### Expression and purification of anti-Fab nanobody

*E. coli*. NiCo21 (DE3) was transformed with the plasmid of the anti-Fab nanobody. Overexpression was induced by 200 μM isopropyl-β-d-thiogalactoside (IPTG) when the cell density reached OD_600_ = 0.6 for 16 h at 16 °C in LB medium. The nanobody was extracted by osmotic shock from the periplasmic space. In brief, the cell pellet was resuspended with hyperosmotic buffer (50 mM Tris (pH 7.5), 20% sucrose, and 0.5 mM EDTA (pH 8.0)), and stirred at 4 °C for 30 min, then centrifugated at 10,000× *g* for 20 min at 4 °C (Beckman, JA14). The cell pellet was resuspended with hypoosmotic buffer (20 mM Tris (pH 7.5) and 5 mM MgCl_2_), and stirred at 4 °C for 30 min, then centrifuged at 25,954× *g* for 30 min at 4 °C (Beckman JA14). The supernatant was subjected to a Ni-NTA column for affinity chromatography and washed with Washing Buffer 1, 2, and 3 in sequence, then eluted with 300 mM imidazole (pH 6.0) and 25 mM NaCl. The nanobody was diluted, loaded onto SP HP 5 mL column (Cytiva), and eluted with SP-B (20 mM MES (pH 6.0), 1 M NaCl) in a linear gradient using the AKTA pure system (GE Healthcare). The peak fractions were pooled for complex assembly.

### Expression of GFP-M-HBsAg-3CA and purification of HBsAg–Fab complex

The BacMam virus for GFP-M-HBsAg-3CA was generated using Sf9 cells^25^. Freestyle 293 F cells at a density of around 3 × 10^6^ cells mL^–1^ were infected with a 2.5% volume of P2 virus. 10 mM sodium butyrate was added 12 h after infection and the cells were incubated at 37 °C for 48 h before harvest. The cell pellet was washed with TBS (20 mM Tris pH 7.5, 150 mM NaCl), then lysed by sonification in lysis buffer (TBS supplemented with 1 μg mL^–1^ aprotinin, 1 μg mL^–1^ pepstatin, 1 μg mL^–1^ leupeptin, 1 mM phenyl methane sulfonyl fluoride). Cell debris and unbroken cells were removed by centrifugation at 7500× *g* for 20 min. Membrane fractions were pelleted by ultracentrifugation at 185,679× *g* for 1 h at 4 °C (Beckman Ti45) and homogenized in lysis buffer, flash frozen, and stored at –80 °C.

To purify the HBsAg-Fab complex, the cell membrane corresponding to around 1.4 L of cell culture was solubilized with 1% LMNG and 0.1% CHS in lysis buffer for 90 min at 4 °C. The insoluble materials were removed with ultracentrifugation at 147,775× *g* for 40 min at 4 °C (Beckman 50.2 Ti), and the supernatant was supplied with Fab and anti-Fab nanobody to form the complex. The lysate was loaded onto 4 mL Streptactin Beads 4FF (Smart-Lifesciences) column and washed with 30 mL buffer W (20 mM Tris (pH 7.5), 150 mM NaCl, and 0.0058% GDN), followed by 30 mL buffer W supplemented with 10 mM ATP and 2 mM MgCl_2_, and 30 mL buffer W again. The target protein was eluted with 5 mM desthiobiotin, 20 mM Tris (pH 8.0), 40 mM Tris (pH 7.5), 50 mM NaCl, and 0.0058% GDN. The eluate was diluted, loaded onto Q HP 1 mL column (Cytiva), and eluted with a linear gradient of Q-B (20 mM Tris (pH 7.5), 1 M NaCl) on AKTA pure system (GE Healthcare). Eluted protein was concentrated using a 100-kDa cutoff concentrator (Millipore) and further purified by Superose 6 increase (Cytiva) running in a buffer containing 20 mM HEPES (pH 7.41), 50 mM NaCl, and 0.0058% GDN. The peak fractions were analyzed by SDS-PAGE, and fractions corresponding to the complex were pooled, concentrated, and used in cryo-EM sample preparation.

For the HBsAg–Fab_HBC_–Fab_GC1102_ complex, the purification was the same as that for HBsAg–Fab_HBC_ complex, except that Fab_GC1102_ was added before size-exclusion chromatography. The peak fractions were analyzed by SDS-PAGE, and fractions corresponding to the complex were pooled, concentrated, and used in cryo-EM sample preparation.

### Strep-tag pull-down assay

The membrane of cells expressing GFP-M-HBsAg was used in the Strep pull-down assay. Cell membrane containing GFP-M-HBsAg was solubilized and ultracentrifuged as described in HBsAg–Fab complex purification. The supernatant was added with different strep-HA-tagged NAbs (used as bait) and Myc-tagged NAbs (used as prey), then incubated with streptactin resin (SA053100; Smart-Lifesciences) at 4 °C on a rotator for 2 h. The beads were washed with TBSG for four times. Bound proteins were eluted with 50 mM Tris (pH 8.0), 150 mM NaCl, 5 mM desthiobiotin, and 0.0058% GDN.

For western blot, input and pull-down fractions were separated with SDS-PAGE and transferred onto polyvinylidene difluoride membranes. Membranes were blocked using 5% nonfat milk in TBST (20 mM Tris (pH 7.4), 150 mM NaCl, and 0.1% Tween-20) for 1 h at room temperature and incubated with primary antibodies (rabbit anti-HA (3724; CST), rabbit anti-Myc (YN5506; Immunoway), both of antibodies were diluted 5000 times) overnight at 4 °C. Then membranes were incubated with HRP-labeled secondary antibody (31444; Thermo Fisher Scientific, the antibody was diluted 10,000 times) for 1 h at room temperature and developed using High-Sig ECL Western Blotting Substrate (Tanon).

### Cryo-EM sample preparation and data collection

For HBsAg–Fab complexes, purified complexes were concentrated to A_280_ = 6.7 (for HBsAg–Fab_HBC_ complex), A_280_ = 7.38 (for HBsAg–Fab_H006_ complex), and A_280_ = 5.2 (for HBsAg–Fab_HBC_–Fab_GC1102_ complex), respectively. Holey carbon grids (Quantifoil Au 300 mesh, R 0.6/1) were glow-discharged by Solaris advanced plasma system (Gatan) for 120 s using 25% O_2_ and 75% Ar. Aliquots of 3 μL concentrated protein sample were applied on glow-discharged grids and the grids were blotted for 3 s before being plunged into liquid ethane using Vitrobot Mark IV (Thermo Fisher Scientific). Cryo-grids were first screened on a Talos Arctica electron microscope (Thermo Fisher Scientific) operating at 200 kV with a K2 camera (Gatan). The screened grids were subsequently transferred to a Titan Krios electron microscope (Thermo Fisher Scientific) operating at 300 kV with a K3 camera (Gatan) and a GIF Quantum energy filter (Gatan) set to a slit width of 20 eV. For Fab_HBC_, images were automatically collected using EPU (Thermo Fisher Scientific) in super-resolution mode at a nominal magnification of ×105,000, corresponding to a calibrated super-resolution pixel size of 0.417 Å with a preset defocus range from –1.5 to –1.8 μm. Each image was acquired as a 2.57-s movie stack of 32 frames with a dose rate of 20.13 e^–^ Å^–2^ s^–1^, resulting in a total dose of about 52 e^–^ Å^–2^. For Fab_H006_ and Fab_HBC_-Fab_GC1102_ complex, images were automatically collected using EPU (Thermo Fisher Scientific) in super-resolution mode at a nominal magnification of ×81,000, corresponding to a calibrated super-resolution pixel size of 0.5335 Å with a preset defocus range from –1.5 to –1.8 μm. Each image was acquired as a 2.56-s movie stack of 32 frames with a dose rate of 22.36 e^–^ Å^–2^ s^–1^, resulting in a total dose of about 50 e^–^ Å^–2^.

### Cryo-EM image analysis

The image processing workflows are illustrated in Supplementary Figures. Super-resolution movie stacks were collected. Motion-correction, two-fold binning, and dose weighting were performed using MotionCor2^26^. Contrast transfer function (CTF) parameters were estimated with cryoSPARC^27^. Micrographs with ice or ethane contamination and empty carbon were removed manually. Particles were auto-picked using Gautomatch (provided by K. Zhang). All subsequent classification and reconstruction were performed in cryoSPARC^27^ unless otherwise stated. Reference-free 2D classification was performed to remove contaminants. The resulting particles were subjected to 3D classification using the initial models generated from cryoSPARC.

For datasets of HBsAg–Fab complexes, particles were first cleaned using 2D and 3D classifications. The classes that showed secondary structure features were selected and the resulting particles were subjected to non-uniform refinement^28^, which resulted in maps with a resolution of 7.63 Å for the HBsAg–Fab_HBC_ complex, 5.51 Å for the HBsAg–Fab_H006_ complex, and 5.29 Å for the HBsAg–Fab_HBC_–Fab_GC1102_ complex. To further improve the resolution, seed-facilitated 3D classification^29^ was performed. The CTF parameters were re-estimated with Patch CTF and particles were re-picked with Template Picker. Several rounds of seed-facilitated 3D classification using good and biased references or references with resolution gradients were performed. The particles were re-picked with Topaz^30^. Several rounds of seed-facilitated 3D classification were performed. To further clean up the dataset, 3D classification without alignment was performed in RELION 3.1^31^ with resolution gradient references with the mask of Fv+AGL. For the HBsAg–Fab_HBC_ complex, the resulting particles were subjected to local non-uniform refinement in cryoSPARC with the mask of Fv+AGL + TM and Fv+AGL, yielding maps with resolutions of 3.25 Å and 3.20 Å, respectively. For the HBsAg–Fab_H006_ complex, the resulting particles were subjected to local non-uniform refinement with the mask of Fv+AGL, yielding a map with a resolution of 3.13 Å. For the HBsAg–Fab_HBC_–Fab_GC1102_ complex, the resulting particles were subjected to local non-uniform refinement with the mask of Fv+AGL, yielding a map with a resolution of 3.09 Å. All of the resolution estimations were based on a Fourier shell correlation of 0.143 cut-off after correcting the masking effect. The local resolution was estimated with cryoSPARC.

### Model building

For HBsAg–Fab complexes, the initial models of Fab_HBC_, Fab_H006_, and Fab_GC1102_ were generated using SWISS-MODEL^32^. The initial models of the Fv region of Fabs were fitted into the maps using UCSF Chimera^33^ and rebuilt manually in Coot^34^. The model of HBsAg was manually built according to the maps. Model refinement was performed using phenix.real_space_refine in PHENIX^35^. The validation statistics are provided in Supplementary Table S1.

### Sequence alignments

Germline sequences of antibodies were determined using IgBlast^36^ and the somatic hypermutation sites on FR1–FR3 of each NAb were determined accordingly. Sequence alignments were performed using ClustalW and illustrated by BioEdit.

### Quantification and statistical analysis

Global resolution estimations of cryo-EM density maps are based on the 0.143 Fourier Shell Correlation criterion^37^. The local resolution was estimated using cryoSPARC. The number of independent experiments (N) and the relevant statistical parameters for each experiment (such as mean or standard deviation) are described in the figure legends. No statistical methods were used to pre-determine sample sizes.

## Supplementary information

Supplementary Figures and Tables

## Data Availability

Cryo-EM maps and the atomic coordinates of the HBsAg–Fab_HBC_ complex have been deposited in the EMDB and PDB under the ID codes EMDB: EMD-63960 and PDB: 9U9B, respectively. Cryo-EM maps and the atomic coordinates of the HBsAg–Fab_H006_ complex have been deposited in the EMDB and PDB under the ID codes EMDB: EMD-61004 and PDB: 9IYY, respectively. Cryo-EM maps and the atomic coordinates of the HBsAg–Fab_HBC_–Fab_GC1102_ complex have been deposited in the EMDB and PDB under the ID codes EMDB: EMD-61788 and PDB: 9JT1, respectively.
