# Supplementary material for: Structural polymorphism of the antigenic loop in HBV surface antigen dictates binding of diverse neutralizing antibodies
Source: Cell Discov. 2025 Jun 17;11:57. doi: 10.1038/s41421-025-00803-2 (PMC12170907; doi:10.1038/s41421-025-00803-2)
Supplement: Supplementary file 1 — Supplementary Figures and Tables [file 41421_2025_803_MOESM1_ESM.pdf]

Supplementary materials for

# Structural Polymorphism of the Antigenic Loop in HBV Surface Antigen Dictates Binding of Diverse Neutralizing Antibodies

Xiao He, Weiyu Tao, Yunlu Kang, Jiaxuan Xu, Xiaoyu Liu, and Lei Chen*

*Correspondence: Lei Chen, chenlei2016@pku.edu.cn

This PDF file includes:

Figs. S1 to S11

Table S1 to S4

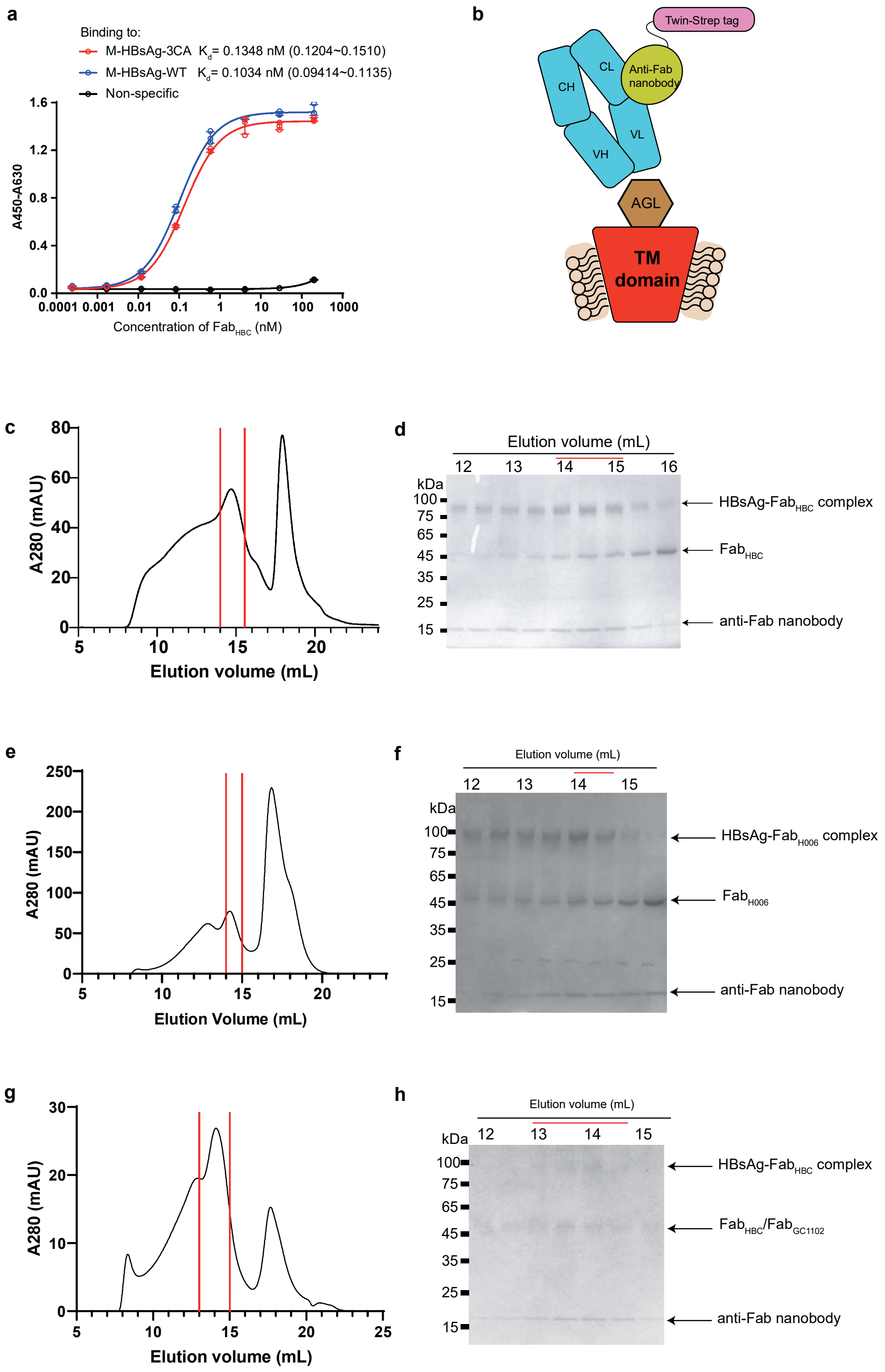


**Figure S1. Protein expression and purification**

**(a)** Binding of Fab$_{HBC}$ to wild-type GFP-tagged M-HBsAg or GFP-tagged M-HBsAg-3CA mutant. The estimated binding affinity is 0.1034 nM (WT M-HBsAg) and 0.1348 nM (M-HBsAg-3CA). An unrelated protein, GFP-tagged anti-ALFA nanobody, was used as the negative control. Data are shown as mean ± standard deviations, *n* = 3 technical replicates. The experiment was performed three times with similar results.

**(b)** Schematic diagram of the assembled HBsAg-Fab complex. Affinity chromatography was performed by using the twin strep tag on the anti-Fab nanobody.

**(c)** Size-exclusion chromatography of the HBsAg-Fab$_{HBC}$ complex on a Superose 6 increase column. The fractions between two red lines were pooled and concentrated for cryo-EM sample preparation.

**(d)** Coomassie brilliant blue staining of SDS-PAGE of fractions from size-exclusion chromatography in **(c)**. The bands corresponding to HBsAg-Fab$_{HBC}$ complex, Fab$_{HBC}$, and anti-Fab nanobody were indicated. The fractions indicated by the red line were used for cryo-EM sample preparation.

**(e)** Size-exclusion chromatography of the HBsAg-Fab$_{H006}$ complex on a Superose 6 increase column. The fractions between two red lines were pooled and concentrated for cryo-EM sample preparation.

**(f)** Coomassie brilliant blue staining of SDS-PAGE of fractions from size-exclusion chromatography in **(e)**. The bands corresponding to HBsAg-Fab$_{H006}$ complex, Fab$_{H006}$, and anti-Fab nanobody were indicated. The fractions indicated by the red line were used for cryo-EM sample preparation.

**(g)** Size-exclusion chromatography of the HBsAg-Fab$_{HBC}$-Fab$_{GC1102}$ complex on a Superose 6 increase column. The fractions between two red lines were pooled and concentrated for cryo-EM sample preparation.

**(h)** Coomassie brilliant blue staining of SDS-PAGE of fractions from size-exclusion chromatography in **(g)**. The bands corresponding to HBsAg-Fab$_{HBC}$ complex, Fab$_{HBC}$, Fab$_{GC1102}$, and anti-Fab nanobody were indicated. The fractions indicated by the red line were used for cryo-EM sample preparation.

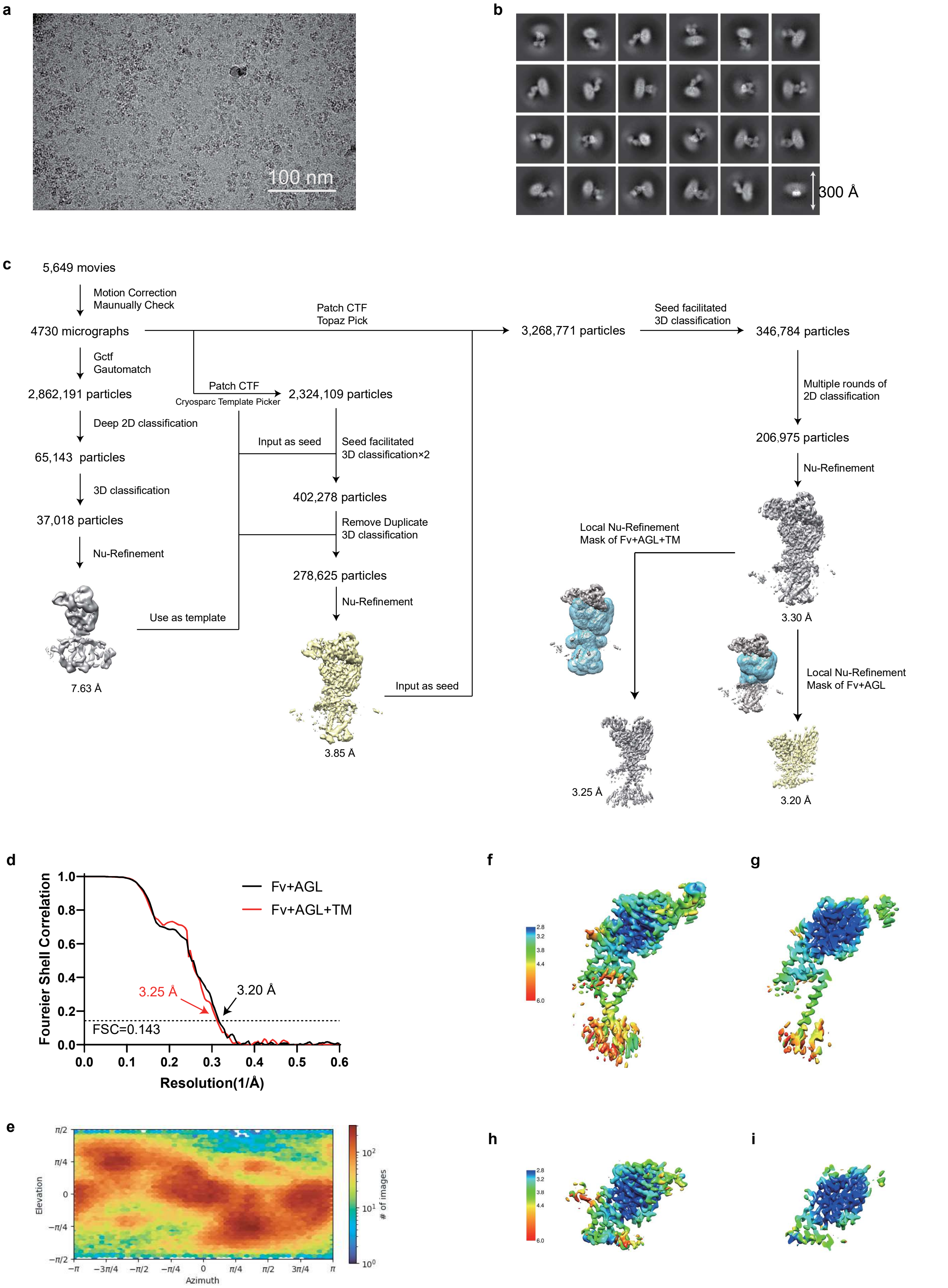


**Figure S2. Cryo-EM data collection and image processing of the HBsAg-Fab$_{HBC}$ complex**

**(a)** Representative raw micrograph (5,649 in total) of HBsAg-Fab$_{HBC}$ complex. Scale bar, 100 nm.

**(b)** 2D-class averages of HBsAg-Fab$_{HBC}$ complex. Scale bar, 300 Å.

**(c)** Cryo-EM data processing workflow of HBsAg-Fab$_{HBC}$ complex. For details, see ‘Cryo-EM image analysis’ in the Methods section.

**(d)** Gold-standard Fourier Shell Correlation (FSC) of the local-refined map shown in **(c)** after correction of masking effects of two different masks.

**(e)** Angular distribution of the final reconstruction of the density maps.

**(f)** Local resolution of HBsAg-Fab$_{HBC}$ complex after refinement using mask Fv+AGL+TM. Scale bar, 2.8-6.0 Å.

**(g)** Cut-open view of **(f)**.

**(h)** Local resolution of HBsAg-Fab$_{HBC}$ complex after refinement using mask Fv+AGL. Scale bar, 2.8-6.0 Å

**(i)** Cut-open view of **(h)**.

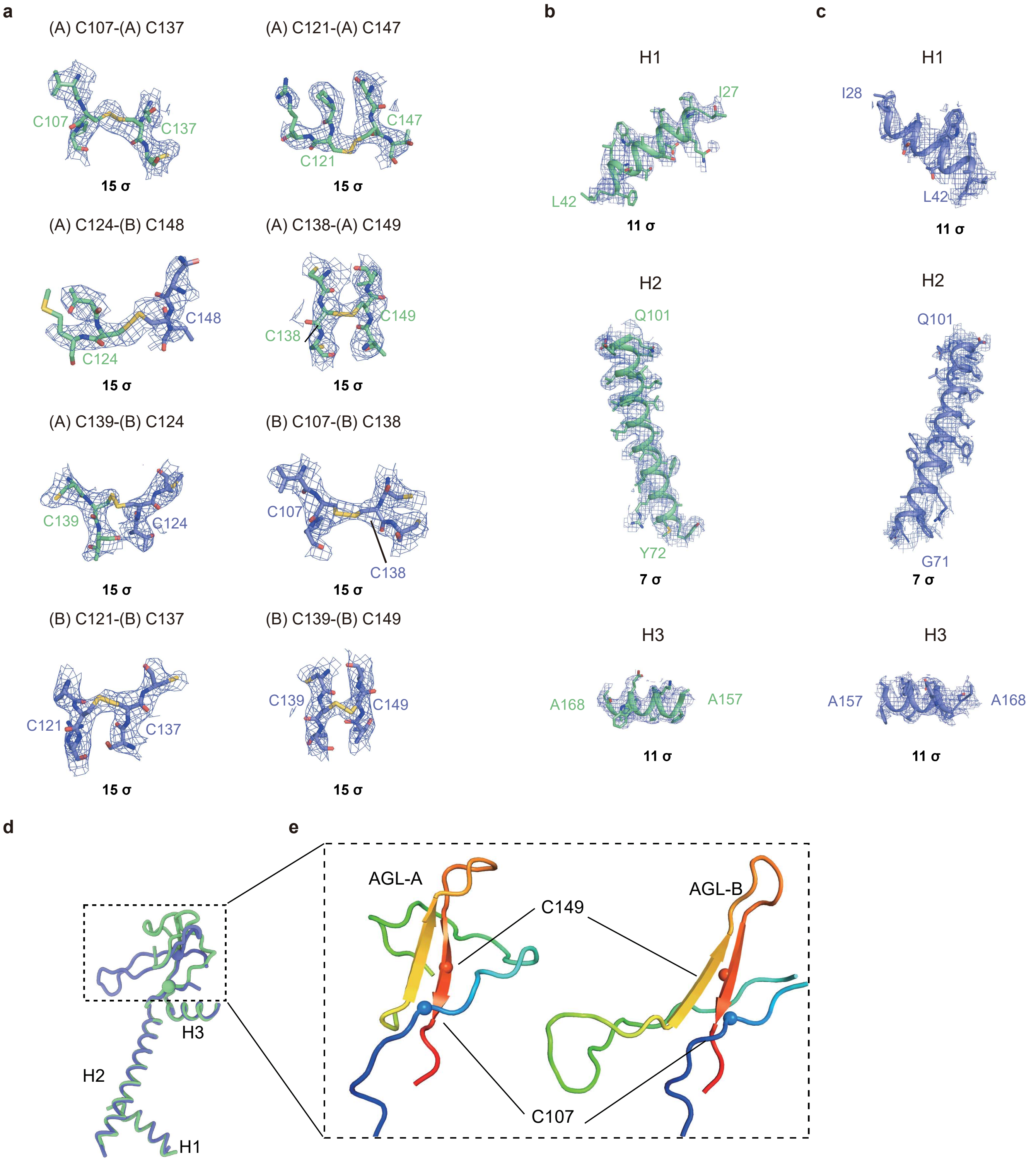


**Figure S3. The electron densities of the asymetry disulfide bond linkages on $AGL_{Type\ A}$**

**(a)** Electron density maps of disulfide bonds in the AGL domain are shown in blue meshes. Contour levels of maps are labeled.

**(b)** Electron density maps of helices in HBsAg-A are shown in blue meshes. Contour levels of maps are labeled.

**(c)** Electron density maps of helices in HBsAg-B are shown in blue meshes. Contour levels of maps are labeled.

**(d)** Structural comparison of Chain A and B in $HBsAg_{Type\ A}$ aligned by TM regions. The Cα of residues where symmetry breaks are denoted as spheres. The AGL domain is indicated with a dashed box.

**(e)** Close-up view of AGL region boxed in **(d)**. AGL-A and AGL-B are shown side-by-side colored in a rainbow with the N-terminus in blue and the C-terminus in red. The Cα of residues where symmetry breaks are indicated.

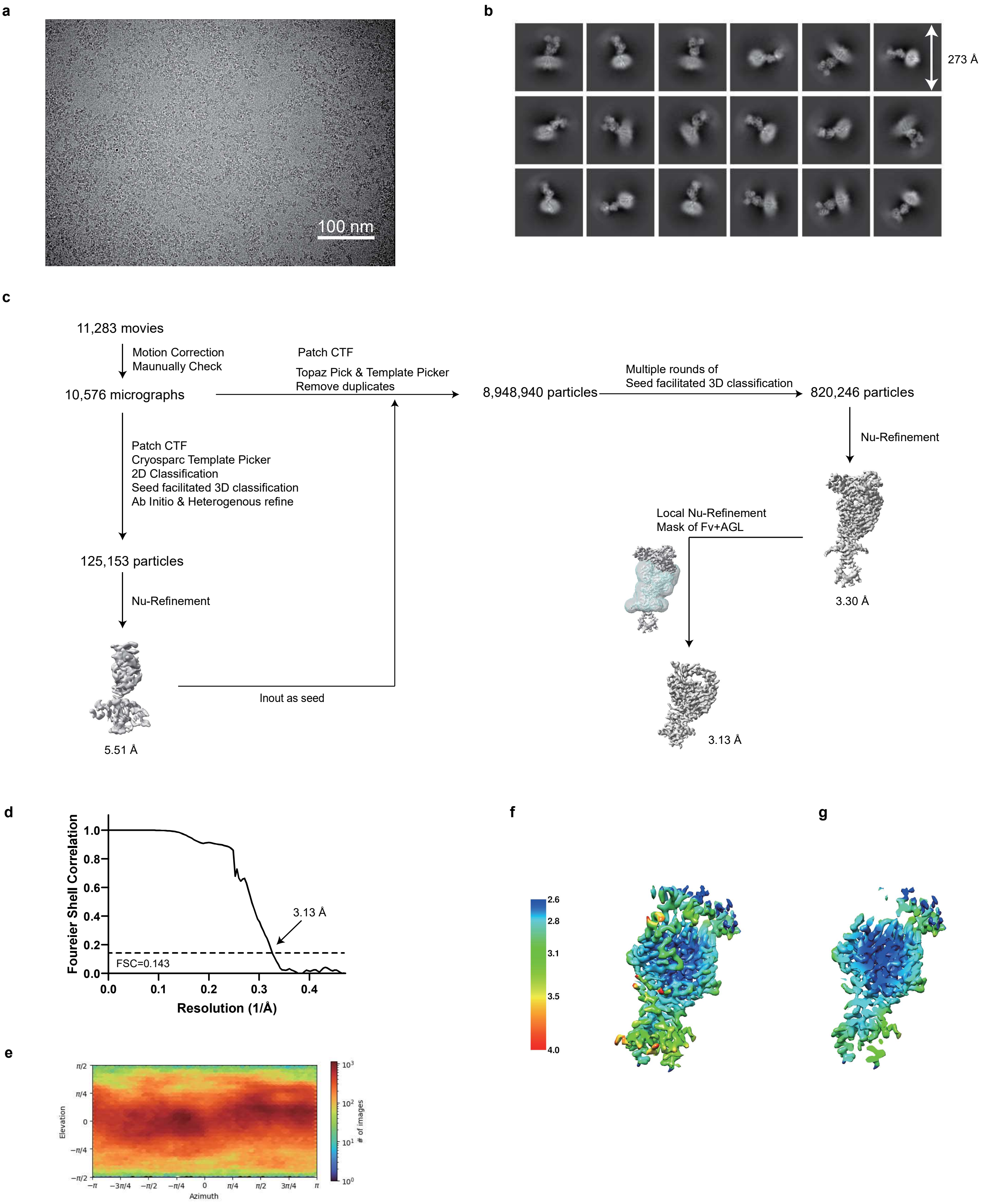


**Figure S4. Cryo-EM data collection and image processing of the HBsAg-Fab$_{H006}$ complex**

**(a)** Representative raw micrograph (11,283 in total) of HBsAg-Fab$_{H006}$ complex. Scale bar, 100 nm.

**(b)** 2D-class averages of HBsAg-Fab$_{H006}$ complex. Scale bar, 273 Å.

**(c)** Cryo-EM data processing workflow of HBsAg-Fab$_{H006}$ complex. For details, see ‘Cryo-EM image analysis’ in the Methods section.

**(d)** Gold-standard Fourier Shell Correlation (FSC) of the local-refined map shown in **(c)** after correction of masking effects.

**(e)** Angular distribution of the final reconstruction of the density map.

**(f)** Local resolution distribution of HBsAg-Fab$_{H006}$ complex. Scale bar, 2.6-4.0 Å.

**(g)** Cut-open view of **(f)**.

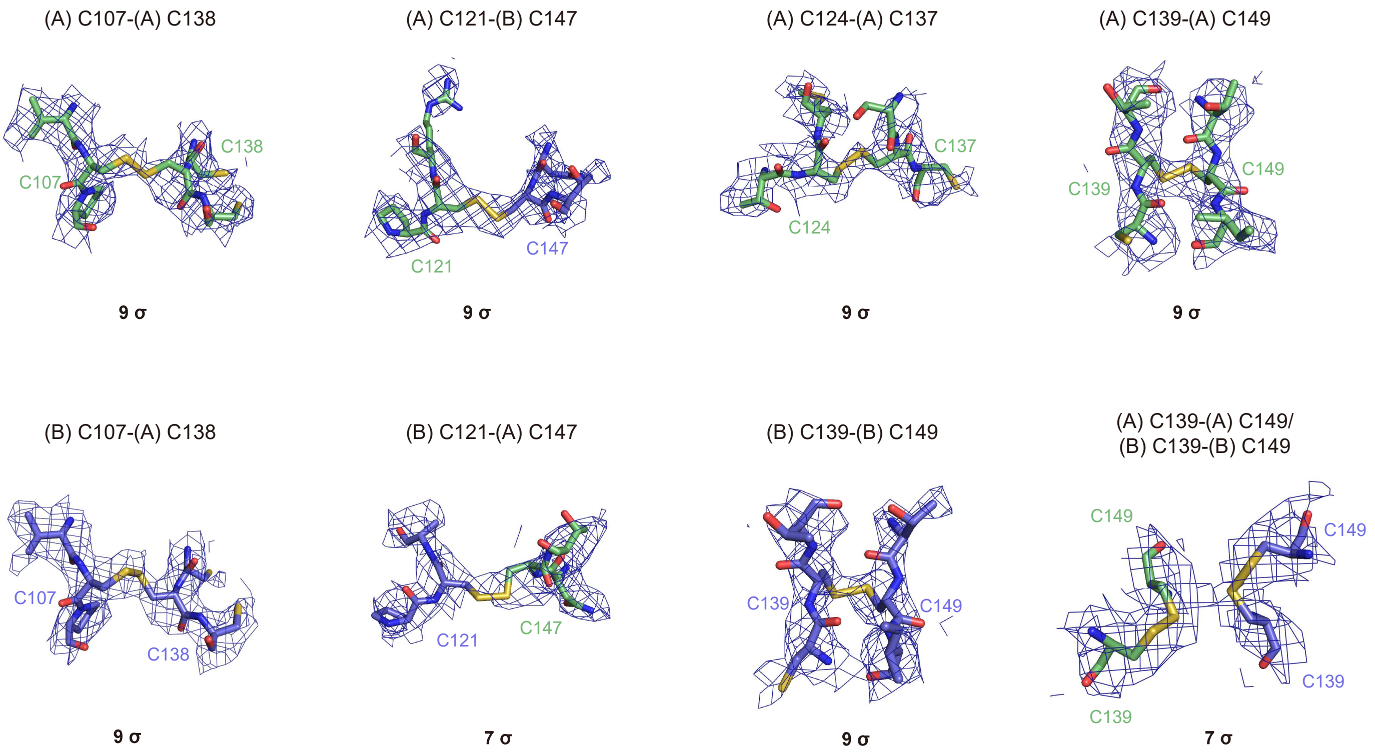


**Figure S5. The electron densities of the disulfide bond linkages on AGL$_{\text{Type B}}$**
Electron density maps of disulfide bonds in the AGL domain are shown in blue meshes. Contour levels of maps are labeled.

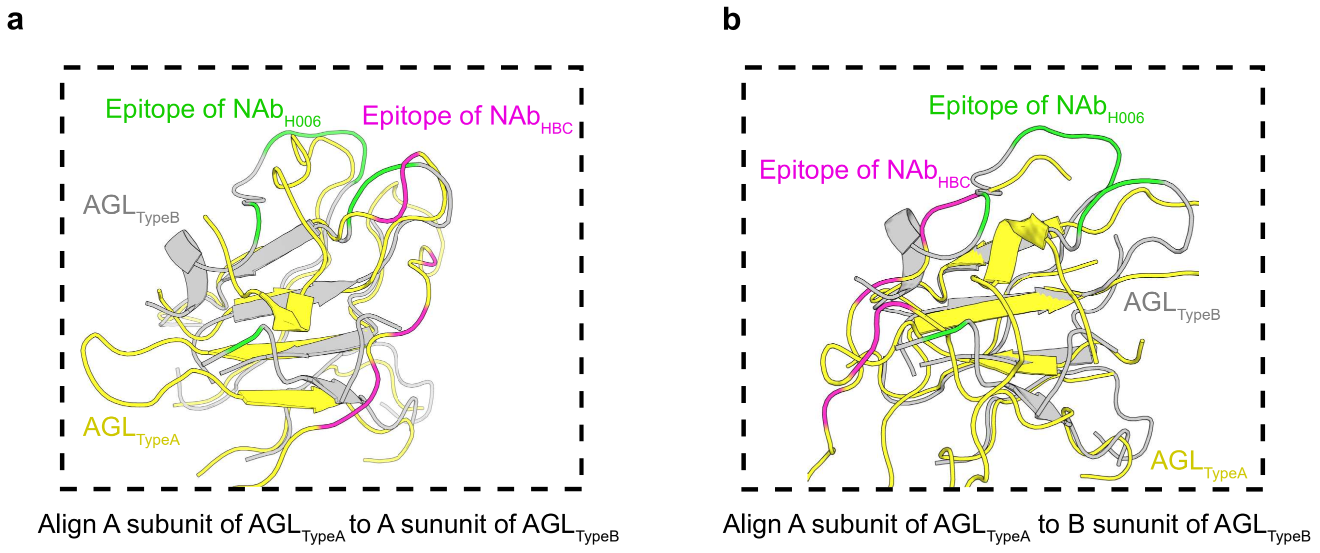


**Figure S6. Structural differences between epitope of $NAb_{HBC}$ and $NAb_{H006}$**

**(a)** Structrual alignment of the subunit A of $AGL_{Type\ A}$ (yellow) onto the subunit A of $AGL_{Type\ B}$ (gray). Structural epitope of $NAb_{HBC}$ on $AGL_{Type\ A}$ is colored in purple. Structural epitope of $NAb_{H006}$ on $AGL_{Type\ B}$ is in green. Large structural differences on the epitopes are observed.

**(b)** Structrual alignment of subunit B of $AGL_{Type\ A}$ (yellow) onto subunit A of $AGL_{Type\ B}$ (gray). Structural epitope of $NAb_{HBC}$ on $AGL_{Type\ A}$ is colored in purple. Structural epitope of $NAb_{H006}$ on $AGL_{Type\ B}$ is in green. Large structural differences on the epitopes are observed.

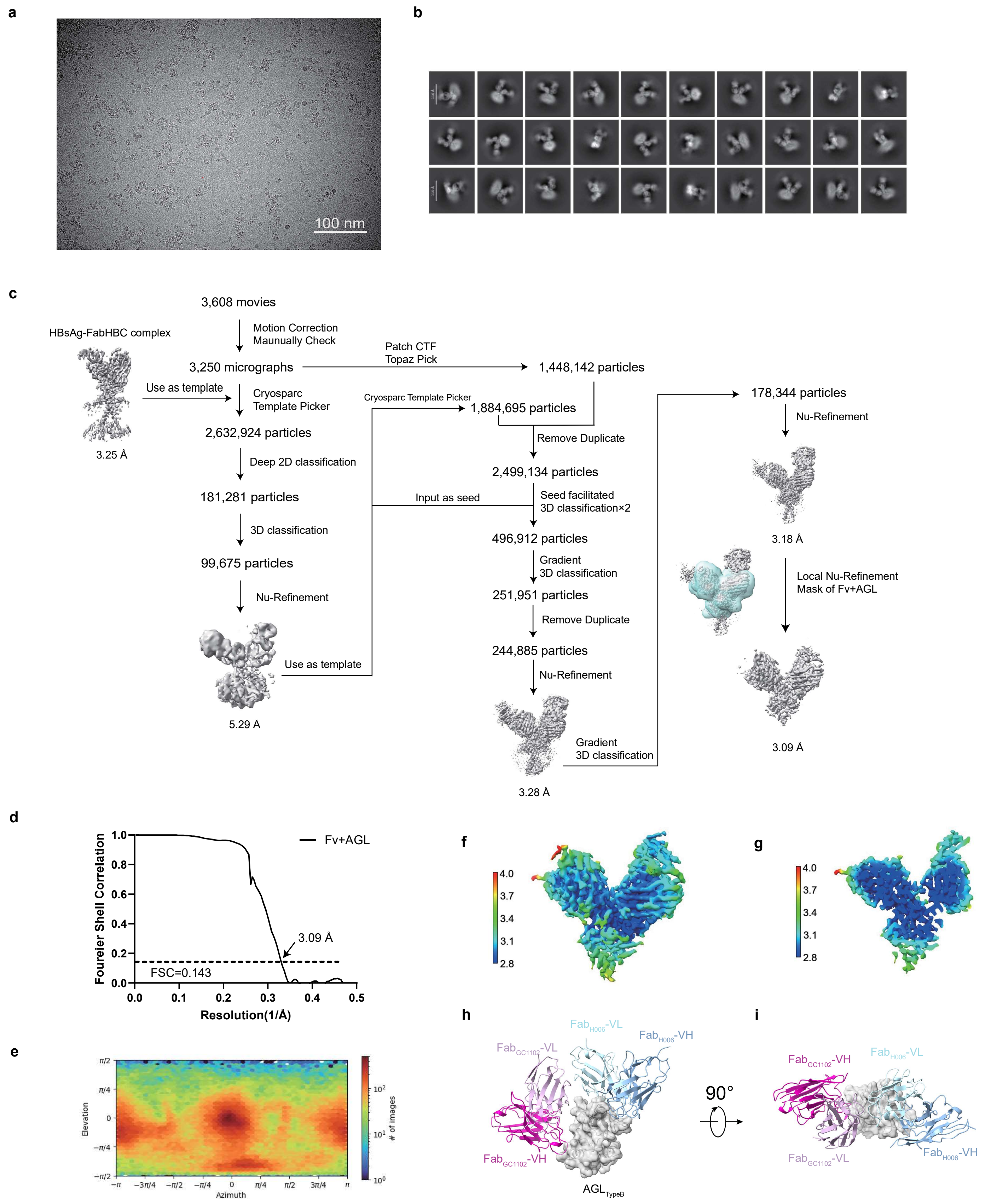


**Figure S7. Cryo-EM data collection and image processing of the HBsAg-Fab$_{HBC}$-Fab$_{GC1102}$ complex**

**(a)** Representative raw micrograph (5,649 in total) of HBsAg-Fab$_{HBC}$-Fab$_{GC1102}$ complex. Scale bar, 100 nm.

**(b)** 2D-class averages of HBsAg-Fab$_{HBC}$-Fab$_{GC1102}$ complex.

**(c)** Cryo-EM data processing workflow of HBsAg-Fab$_{HBC}$-Fab$_{GC1102}$ complex. For details, see 'Cryo-EM image analysis' in the Methods section.

**(d)** Gold-standard Fourier Shell Correlation (FSC) of the local-refined map shown in **(c)** after correction of masking effects.

**(e)** Angular distribution of the final reconstruction of the density maps.

**(f)** Local resolution of HBsAg-Fab$_{HBC}$-Fab$_{GC1102}$ complex after refinement using mask Fv+AGL. Scale bar, 2.8-4.0 Å.

**(g)** Cut-open view of **(f)**.

**(h)** The structure of Fab$_{GC1102}$ is aligned to that of HBsAg in complex of Fab$_{H006}$, using H3 of HBsAg as the reference. No sterical clash between Fab$_{GC1102}$ (purple) and Fab$_{H006}$ (blue) are observed.

**(i)** A 90°-rotated view of **(h)**.

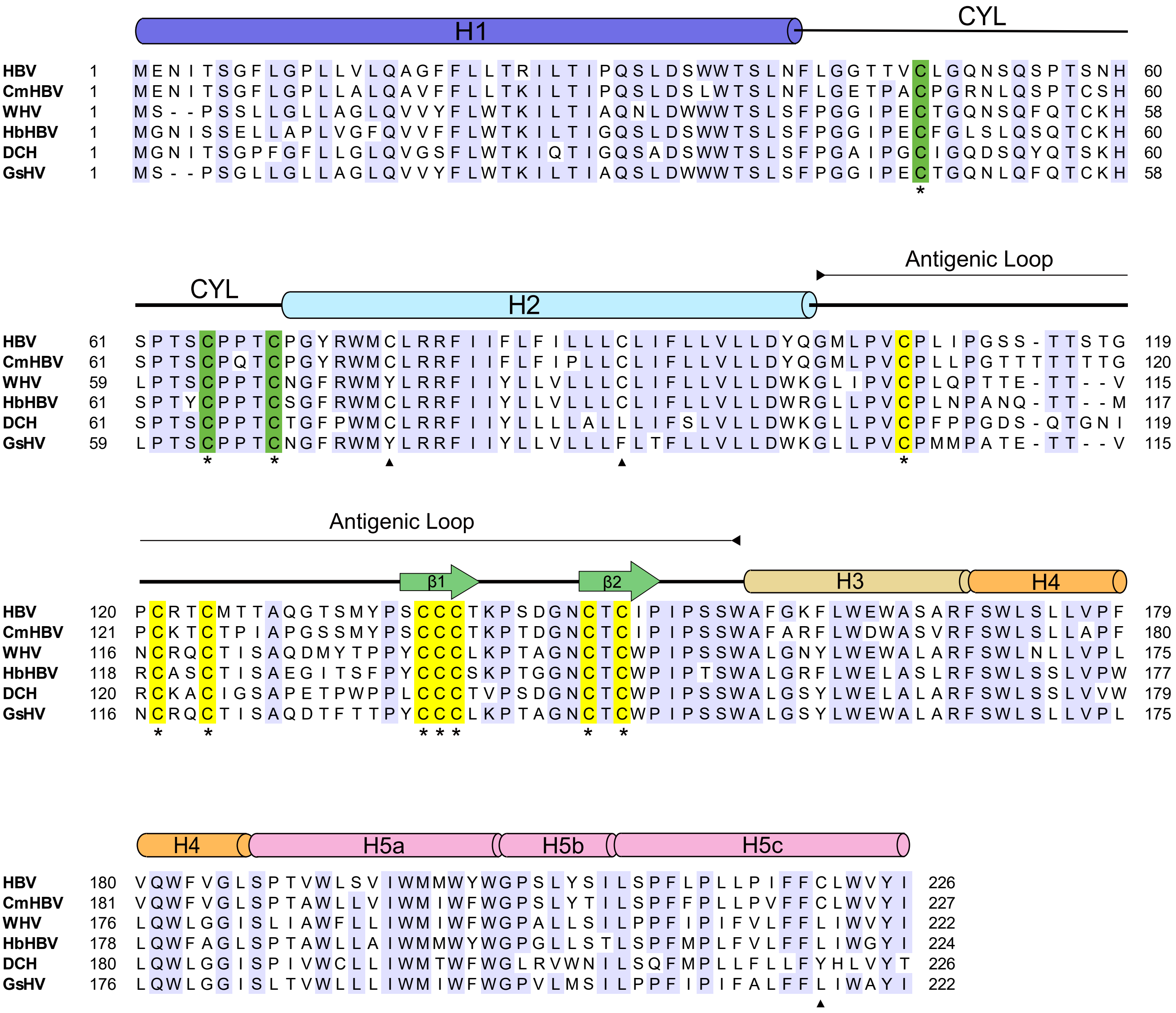


**Figure S8. Sequence alignment of the surface antigens of several HBV-related viruses**

Multiple sequence alignment (MSA) of S-HBsAg sequences of Hepatitis B virus (used in experiment, RefSeq: YP_009173871.1), Capuchin monkey hepatitis B virus (CmHBV, RefSeq: YP_009666526.1), Woodchuck hepatitis virus (WHV, RefSeq: NP_944491.1), Horseshoe bat hepatitis B virus (HbHBV, RefSeq: YP_009045996.1), Domestic cat hepadnavirus (DCH, RefSeq: YP_009553237.1) and Ground squirrel hepatitis virus (GsHV, RefSeq: NP_955537.1). The sequences are downloaded from NCBI. α helices and β sheets are shown as cylinders and wide arrows respectively. Conserved cysteines are denoted with asterisks, among which those localized on AGL are highlighted in yellow, and those on CYL are marked in green. Other conserved residues are indicated in light purple. Three cysteines (HBV C76, C90, and C221) mutated in M-HBsAg-3CA are indicated with black triangles.

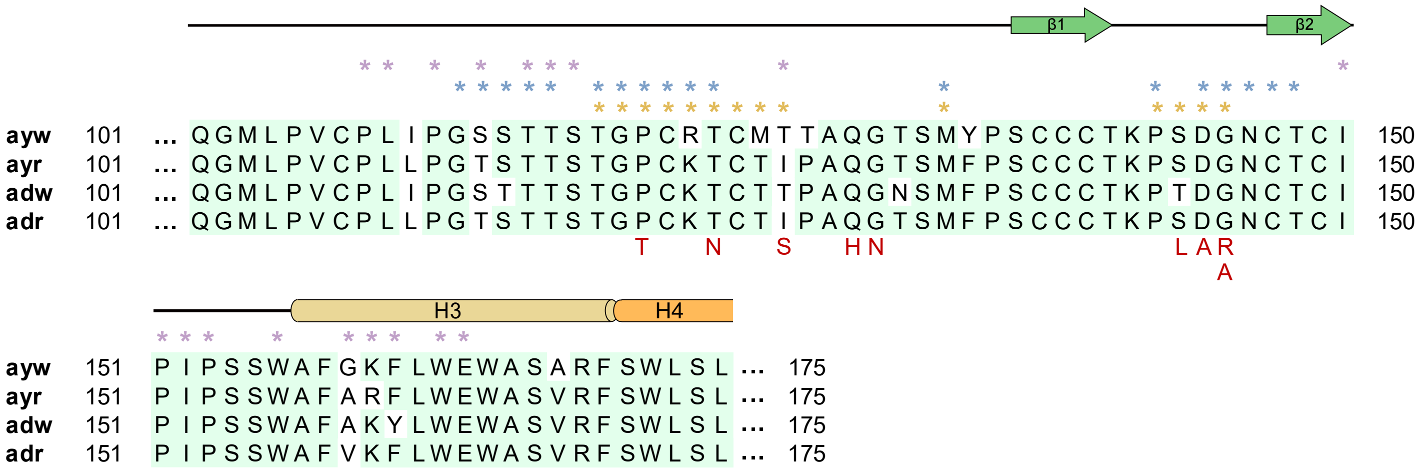

**Figure S9. Sequence alignment of different HBsAg serotypes**

MSA of representative sequences of HBsAg serotype ayw (used in experiment, RefSeq: YP_009173871.1), ayr (GenBank: AAA45525.1), adw (GenBank: ABC02228.1), and adr (GenBank: AAA45522.1). The sequences are downloaded from NCBI. α helices and β sheets are shown as cylinders and wide arrows respectively. Conserved residues were indicated in light green. Residues comprising epitopes of NAbs were indicated with asterisks (for $NAb_{HBC}$ in orange, for $NAb_{H006}$ in steel blue, and for $NAb_{GC1102}$ in light purple, respectively) above the sequences. Frequently observed escape mutations were indicated with red letters below the sequences . Sequence alignment was performed with ClustalW and illustrated by BioEdit.

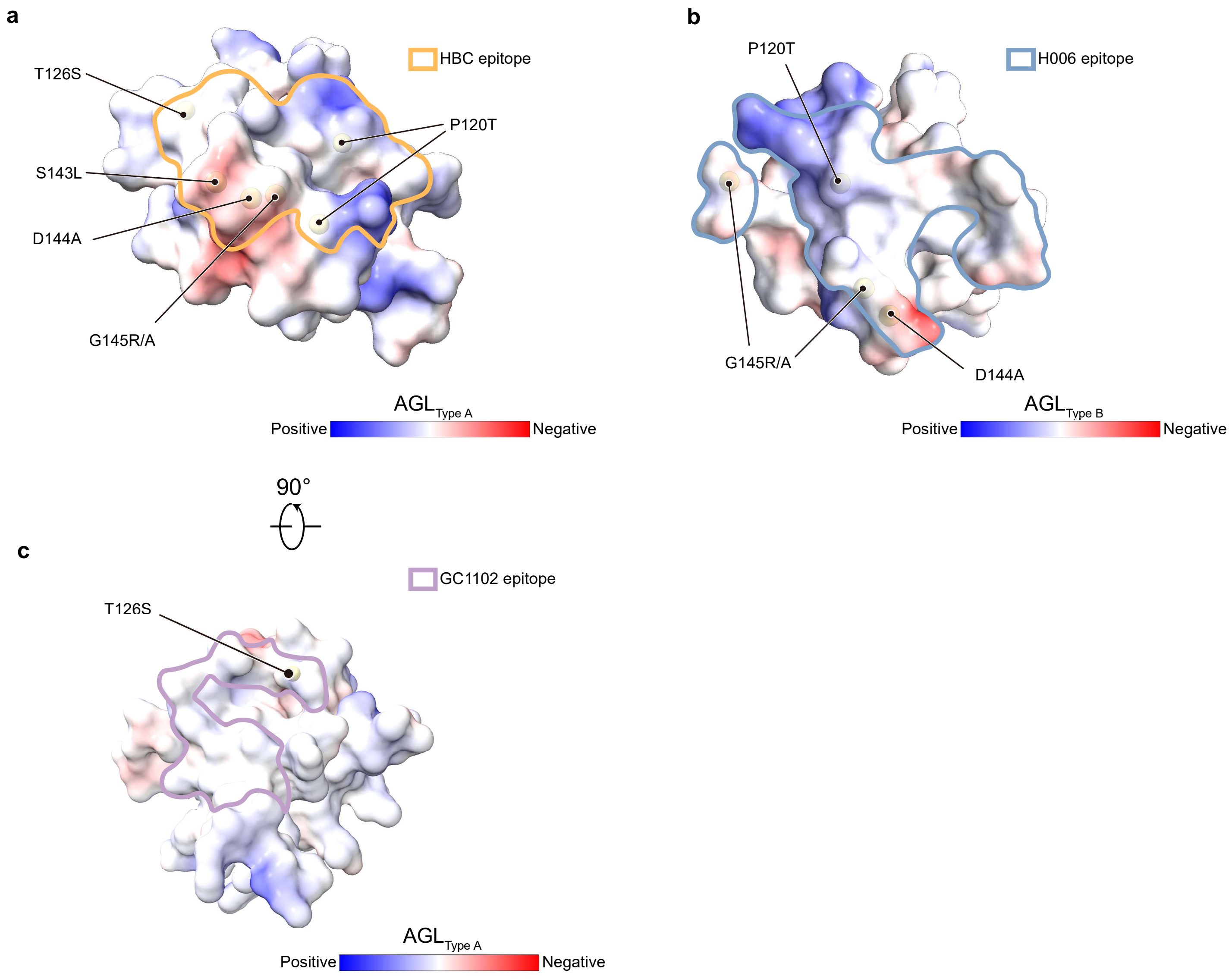


**Figure S10. Spatial distribution of frequently observed HBV escape mutations on HBsAg**
**(a)** Semi-transparent surface representation of $AGL_{Type\ A}$, colored by electrostatic potential. The epitope of $NAb_{HBC}$ was circled in an orange line. Frequently observed escape mutation sites on this epitope were indicated with yellow spheres.
**(b)** Semi-transparent surface representation of $AGL_{Type\ B}$, colored by electrostatic potential. The epitope of $NAb_{H006}$ was circled in a blue line. Frequently observed escape mutation sites on this epitope were indicated with yellow spheres.
**(c)** A 90°-rotated view of surface representation in **(a)**, with the epitope of $NAb_{GC1102}$ circled in a purple line. Frequently observed escape mutation sites on this epitope were indicated with yellow spheres.

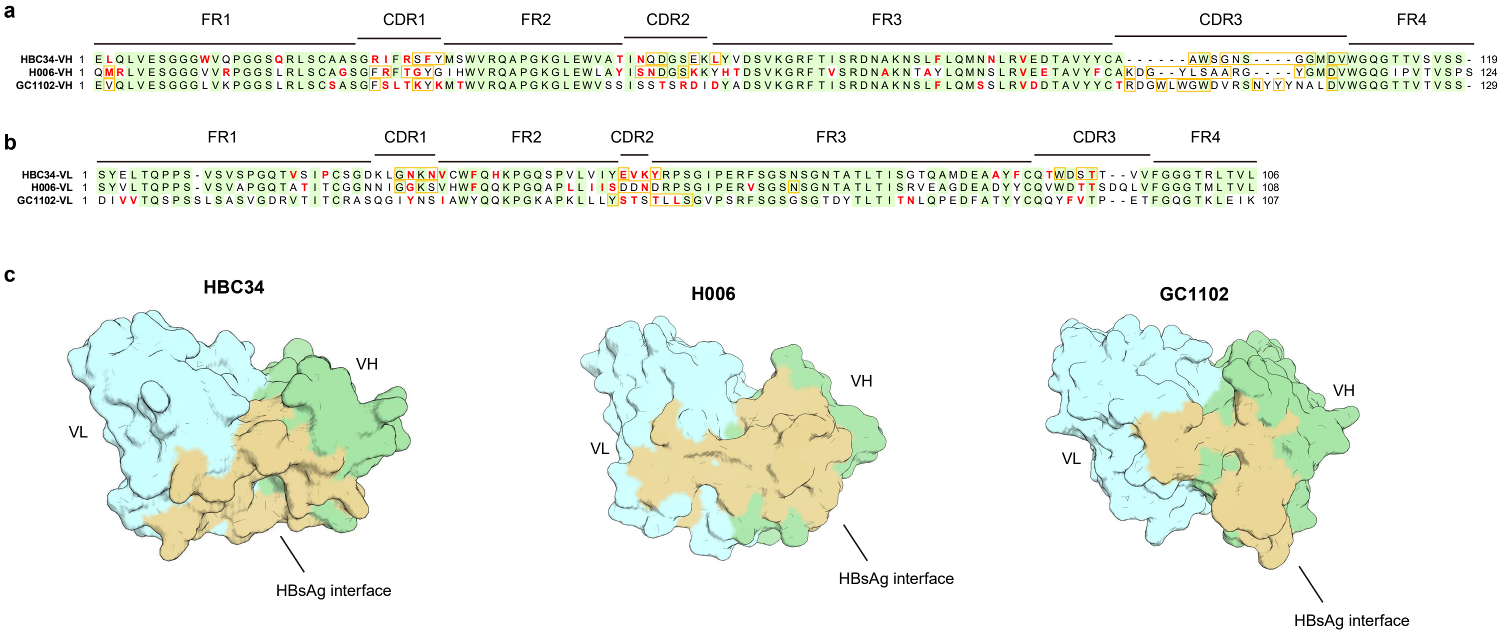


**Figure S11. Paratopes of HBV NAbs**

**(a)** Alignment of VH regions of $NAb_{HBC}$, $NAb_{H006}$ and $NAb_{GC1102}$. Identical residues are indicated in light green. Boxed residues in orange lines compose HBsAg interfaces of NAbs. Antibody germlines were determined using IgBlast, which for $NAb_{HBC}$ was IGHV3-7, for $NAb_{H006}$ was IGHV3-30, and for $NAb_{GC1102}$ was IGHV3-21, respectively, and the somatic hypermutation sites on FR1-FR3 of each NAb are highlighted in red letters. Sequence alignment was performed with ClustalW and illustrated by BioEdit.

**(b)** Alignment of VL regions of $NAb_{HBC}$, $NAb_{H006}$ and $NAb_{GC1102}$. Identical residues are indicated in light green. Boxed residues in orange lines compose HBsAg interfaces of NAbs. Antibody germlines were determined using IgBlast, which for $NAb_{HBC}$ was IGLV3-1, for $NAb_{H006}$ was IGLV3-21, and for $NAb_{GC1102}$ was IGKV1-NL1, respectively, and the somatic hypermutation sites on FR1-FR3 of each NAb are highlighted in red letters. Sequence alignment was performed with ClustalW and illustrated by BioEdit.

**(c)** Surface representations of Fv regions of $NAb_{HBC}$, $NAb_{H006}$ and $NAb_{GC1102}$, exhibiting the CDRs. Heavy chains are colored in green while light chains are colored in green. HBsAg interfaces are indicated in yellow.

**Table S1. Cryo-EM data collection, refinement, and validation statistics**

The parameters for the Cryo-EM data collection, processing, and validation of HBsAg-Fab complex are listed in the table.

| PDB ID<br>EMDB ID | HBsAg-$Fab_{HBC}$<br>9U9B<br>EMD-63960 | HBsAg-$Fab_{H006}$<br>9IYY<br>EMD-61004 | HBsAg-$Fab_{HBC}$-$Fab_{GC1102}$<br>9JT1<br>EMD-61788 |
|---|---|---|---|
| **Data collection and processing** | | | |
| Magnification | 105,000 × | 81,000 × | 81,000 × |
| Voltage (kV) | 300 | 300 | 300 |
| Electron exposure ($e^-/Å^2$) | 52 | 50 | 50 |
| Defocus range (μm) | -1.5 to -1.8 | -1.5 to -1.8 | -1.5 to -1.8 |
| Pixel size (Å) | 0.834 | 1.067 | 1.067 |
| Symmetry imposed | *C1* | *C1* | *C1* |
| Initial particle images (no.) | 3,268,771 | 8,948,940 | 2,499,134 |
| Final particle images (no.) | 206,975 | 820,246 | 178,344 |
| Map resolution (Å) | 3.25[a]/3.20[b] | 3.13 | 3.09 |
| FSC threshold | 0.143 | 0.143 | 0.143 |
| Map resolution range (Å) | 250-3.25 | 250-3.13 | 250-3.09 |
| | | | |
| **Refinement** | | | |
| Initial model used (PDB code) | Swiss-model (Fab) | Swiss-model (Fab) | Swiss-model (Fab) |
| Model resolution (Å) | 3.96 | 3.23 | 3.22 |
| FSC threshold | 0.5 | 0.5 | 0.5 |
| Model resolution range (Å) | 250-3.96 | 250-3.23 | 250-3.22 |
| Map sharpening *B* factor ($Å^2$) | -124.5[a]/-117.0[b] | -176.2 | -152.2 |
| Model composition | | | |
| Non-hydrogen atoms | 3,261 | 2,534 | 4589 |
| Protein residues | 439 | 342 | 609 |
| Ligands | 0 | 0 | 0 |
| *B* factors ($Å^2$) | | | |
| Protein | 61.86 | 21.72 | 82.06 |
| Ligand | --- | --- | --- |
| R.m.s. deviations | | | |
| Bond lengths (Å) | 0.004 | 0.004 | 0.003 |
| Bond angles (°) | 1.004 | 0.564 | 0.535 |
| Validation | | | |
| MolProbity score | 1.80 | 2.37 | 1.97 |
| Clashscore | 8.37 | 8.29 | 10.22 |
| Poor rotamers (%) | 0 | 0 | 0 |
| Ramachandran plot | | | |
| Favored (%) | 95.04 | 95.40 | 97.13 |
| Allowed (%) | 4.96 | 4.60 | 2.87 |
| Disallowed (%) | 0 | 0 | 0 |

[a], The values of focused refinement of mask1 (Fv+TM+AGL).

[b], The values of focused refinement of mask2 (Fv+AGL)

**Table S2 Detailed interactions between HBsAg and $Fab_{HBC}$**

The residues involved in polar interactions between HBsAg and $Fab_{HBC}$ and their locations are listed in the table. The notations (N) and (O) indicate whether the interaction is mediated by the main chain nitrogen or oxygen atom of the residue, respectively.

| **HBsAg** | | **$Fab_{HBC}$** | |
|---|---|---|---|
| **Location** | **Residue** | **Location** | **Residue** |
| (A) H2-β1 Loop | R122 | CDRL1 | G28 (O) |
| | | | N29 (O) |
| | | | K30 (O) |
| (A) β1-β2 Loop | T140 | CDRL2 | E49 |
| | P142 (O) | CDRL3 | W90 |
| | S143 | CDRH2 | E57 |
| | S143 (O) | CDRL3 | T93 |
| (B) H2-β1 Loop | T123 | CDRH3 | S100 (O) |
| | | | W99 (O) |
| | T126 (O) | CDRH1 | S31 |
| | T126 (N) | | S31 (O) |

**Table S3 Detailed interactions between HBsAg and Fab$_{H006}$**

The residues involved in polar interactions between HBsAg and Fab$_{H006}$ and their locations are listed in the table. The notations (N) and (O) indicate whether the interaction is mediated by the main chain nitrogen or oxygen atom of the residue, respectively.

| **HBsAg** | | **Fab$_{H006}$** | |
|---|---|---|---|
| **Location** | **Residue** | **Location** | **Residue** |
| (A) β1-β2 Loop | G145 (O) | CDRL2 | N51 |
| (B) H2-β1 Loop | G112 (N) | CDRH3 | S103 |
| | S114 (N) | CDRH2 | D54 |
| | S114 (O) | CDRH3 | S103 |
| | T115 | CDRH2 | N53 |
| | | | D54 |
| | | | S56 |
| | T116 (N and side chain) | CDRH3 | S103 (O) |
| | T116 (O) | | A105 (N) |
| | T118 | | L102 (O) |
| | R122 | CDRL1 | G28 (O) |
| | T123 | | S31 |
| (B) β1-β2 Loop | D144 | CDRH1 | R28 (N) |
| | | | Y32 |
| | D144 (O) | CDRH3 | K98 |
| | N146 (N) | | G100 (O) |
| | T148 | | L102 (N) |

**Table S4 Detailed interactions between HBsAg and Fab$_{GC1102}$**

The residues involved in polar interactions between HBsAg and Fab$_{GC1102}$ and their locations are listed in the table. The notations (N) and (O) indicate whether the interaction is mediated by the main chain nitrogen or oxygen atom of the residue, respectively.

| **HBsAg** | | **Fab$_{GC1102}$** | |
|---|---|---|---|
| **Location** | **Residue** | **Location** | **Residue** |
| (A) H2-β1 Loop | S113 | CDRH3 | R98 |
| | S113 (O and side chain) | CDRH1 | Y32 |
| | T115 (O) | CDRL2 | S56 (N) |
| | T116 | | Y49 |
| | S117 (N) | | L54 (O) |
| (A) β2-H3 Loop | P151 (O) | CDRH3 | Y112 |
| (A) H3 | K160 | | N110 |
| | E164 | CDRH1 | K31 |
| | | CDRH3 | G104 (N) |
